# Healthy Lung Vessel Morphology Derived From Thoracic Computed Tomography

**DOI:** 10.3389/fphys.2018.00346

**Published:** 2018-04-10

**Authors:** Michael Pienn, Caroline Burgard, Christian Payer, Alexander Avian, Martin Urschler, Rudolf Stollberger, Andrea Olschewski, Horst Olschewski, Thorsten Johnson, Felix G. Meinel, Zoltán Bálint

**Affiliations:** ^1^Ludwig Boltzmann Institute for Lung Vascular Research, Graz, Austria; ^2^Clinic and Policlinic of Radiology, Ludwig-Maximilians-University Hospital, Munich, Germany; ^3^Faculty of Computer Science and Biomedical Engineering, Institute of Computer Graphics and Vision, Graz University of Technology, Graz, Austria; ^4^Institute for Medical Informatics, Statistics and Documentation, Medical University of Graz, Graz, Austria; ^5^Ludwig Boltzmann Institute for Clinical-Forensic Imaging, Graz, Austria; ^6^Faculty of Computer Science and Biomedical Engineering, Institute of Medical Engineering, Graz University of Technology, Graz, Austria; ^7^Division of Pulmonology, Department of Internal Medicine, Medical University of Graz, Graz, Austria; ^8^Radiologie München GbR, Munich, Germany; ^9^Department of Diagnostic and Interventional Radiology, Rostock University Medical Center, Rostock, Germany; ^10^Faculty of Physics, Babeş-Bolyai University, Cluj-Napoca, Romania

**Keywords:** pulmonary circulation, morphology, computed tomography, healthy reference values, automated image analysis, artery/vein separation

## Abstract

Knowledge of the lung vessel morphology in healthy subjects is necessary to improve our understanding about the functional network of the lung and to recognize pathologic deviations beyond the normal inter-subject variation. Established values of normal lung morphology have been derived from necropsy material of only very few subjects. In order to determine morphologic readouts from a large number of healthy subjects, computed tomography pulmonary angiography (CTPA) datasets, negative for pulmonary embolism, and other thoracic pathologies, were analyzed using a fully-automatic, in-house developed artery/vein separation algorithm. The number, volume, and tortuosity of the vessels in a diameter range between 2 and 10 mm were determined. Visual inspection of all datasets was used to exclude subjects with poor image quality or inadequate artery/vein separation from the analysis. Validation of the algorithm was performed manually by a radiologist on randomly selected subjects. In 123 subjects (men/women: 55/68), aged 59 ± 17 years, the median overlap between visual inspection and fully-automatic segmentation was 94.6% (69.2–99.9%). The median number of vessel segments in the ranges of 8–10, 6–8, 4–6, and 2–4 mm diameter was 9, 34, 134, and 797, respectively. Number of vessel segments divided by the subject's lung volume was 206 vessels/L with arteries and veins contributing almost equally. In women this vessel density was about 15% higher than in men. Median arterial and venous volumes were 1.52 and 1.54% of the lung volume, respectively. Tortuosity was best described with the sum-of-angles metric and was 142.1 rad/m (138.3–144.5 rad/m). In conclusion, our fully-automatic artery/vein separation algorithm provided reliable measures of pulmonary arteries and veins with respect to age and gender. There was a large variation between subjects in all readouts. No relevant dependence on age, gender, or vessel type was observed. These data may provide reference values for morphometric analysis of lung vessels.

## Introduction

Morphometric information on the lung vasculature in healthy subjects is necessary to improve our understanding about the complex and interconnected functional network of the lung and to recognize pathologic deviations beyond the normal variation expected between subjects (Weibel, 2009, 2017). The established normal values for number and size of lung vessels have been derived from vessel casts of necropsy material (Singhal et al., 1973; Horsfield, 1978; Horsfield and Gordon, 1981; Huang et al., 1996). These studies quantified lung vessels in detail, but were based on very few subjects and arteries and veins were analyzed from different subjects. Moreover, the preparation of the vessel casts required a higher filling pressure compared to physiological pressures in man (Kovacs et al., 2009). These limitations underpin the need for *in vivo* analysis of lung vessel morphology where, within a large cohort of healthy subjects, the range of normal values can be determined and the arterial and venous vessel morphology can be directly compared within the same subject.

Radiologic evaluation of lung vasculature in thoracic computed tomography (CT) images allows the description of dilatation, tortuosity, and tapering of the pulmonary vessels down to diameters of about 1 mm (Resten et al., 2005; Marano et al., 2015). The large amount of vessels dispersed throughout the lung and the long time necessary for manually labeling them precludes manual quantification of all arteries and veins by a human reader. Computational algorithms can identify lung vessels from the thoracic CT images in an observer independent manner and deliver morphometric readouts of the lung vessels (van Rikxoort and van Ginneken, 2013). The information on lung vessel morphology in a large cohort of healthy subjects can provide important information on age- and gender-dependent structural changes in the vasculature (Lopes et al., 2012; Novella et al., 2012). Further, quantitative measures of the lung vessel morphology were shown to have diagnostic and/or prognostic value in diseases like pulmonary hypertension (Matsuoka et al., 2010b; Helmberger et al., 2014; Rahaghi et al., 2016a), chronic obstructive pulmonary disease (COPD) (Matsuoka et al., 2010a; Estépar et al., 2013; Rahaghi et al., 2016b), or idiopathic pulmonary fibrosis (IPF) (Jacob et al., 2016, 2017). For these applications it is important to know the normal ranges of the morphologic readouts (Li et al., 2003, 2012; Hansell, 2010; Hoffman et al., 2014).

Exposure to ionizing radiation cannot be justified in a relevant number of healthy volunteers from different gender and age groups. Therefore, we used CT investigations that had been performed for suspicion of acute pulmonary embolism but with a negative result (Meinel et al., 2013). Since these patients may have other pathologies, they were examined for anomalies and pathologies of the lung, heart, or skeleton, as obtained from the thoracic CT scans or diagnoses from the patient history. Patients with any detected pathologies were excluded from this study.

Recently, we presented an in-house developed fully-automatic algorithm for artery/vein separation in thoracic CT images (Payer et al., 2016). This algorithm identifies pulmonary arteries and veins with high accuracy and determines morphologic measures of the individual vessel segments. The user interaction is limited to a visual inspection to ascertain a sufficient quality of the final segmentation. Thus, a highly accurate artery/vein labeling can be achieved with little manual effort and the analysis of large numbers of subjects becomes tractable. The use of the algorithm further allows evaluation of the distribution of morphologic readouts within a subject.

The aim of the present study was to quantitatively determine pulmonary arterial and venous morphology in a large cohort of subjects with no apparent thoracic abnormalities. We applied the automatic artery/vein separation algorithm and provide reference values of lung vessel morphology together with their normal range in physiologic conditions in relation to age, gender, and lung volume.

## Subjects and methods

This study was carried out in accordance with the recommendations of the Guidelines on the diagnosis and management of acute pulmonary embolism by the Task Force for the Diagnosis and Management of Acute Pulmonary Embolism of the European Society of Cardiology (Torbicki et al., 2008). All subjects provided written informed consent for the computed tomography pulmonary angiography (CTPA) examination in accordance with the Declaration of Helsinki. The ethics committee of the Ludwig-Maximilians-University, Munich, Germany, waived ethical approval and individual informed consent for retrospective data analysis. The CTPA datasets were acquired at Ludwig-Maximilians-University Hospital, Munich, Germany between May 2009 and November 2011. The inclusion criterion was a clinically indicated CTPA, negative for pulmonary embolism. Exclusion criteria were: (i) CTPA with severe artifacts, inadequate vascular enhancement or incomplete datasets; (ii) Any thoracic pathology detected on dual-energy CTPA or known from the clinical history of the respective patient. A total of 1,321 CTPAs for suspected pulmonary embolism (PE) were performed. Of these examinations, 224 were positive for acute PE and 841 showed thoracic pathologies. Further 114 examinations had to be excluded due to severe artifacts, inadequate vascular enhancement, or incomplete datasets. The remaining 142 subjects met all eligibility criteria. Four subjects had to be excluded à *posteriori* because of motion artifacts that precluded automatic image analysis. For 2 further subjects no CT datasets were available at the time of data transfer. Thus, 136 subjects were included into this study.

### Examinations

The CTPA images were acquired with a 128-slice dual-source CT scanner (Somatom Definition Flash, Siemens Healthineers, Forchheim, Germany). The detailed description of the imaging protocol is presented in Meinel et al. (2013). Briefly, 85 ml contrast material (Iopromide, Ultravist 370, Bayer Schering Pharma, Berlin, Germany) were administered via an antecubital vein at a flow rate of 5 mL/s, followed by 50 mL of saline injected at the same flow rate. Scan timing was determined using automatic bolus tracking in the *truncus pulmonalis*. Collimation was set to 32 × 0.6 mm with a pitch of 0.5. The images were reconstructed with 1.5 mm slice thickness with 1.0 mm increments using a medium-soft kernel (D30f). The anonymized datasets were transferred to an independent workstation. The images from both detectors (tin-filtered 140 and 100 kVp) were equally mixed and used for the analysis. In addition to the CT datasets, only information on age and gender was available for each subject.

### Data processing and analysis

**Figure 1:**
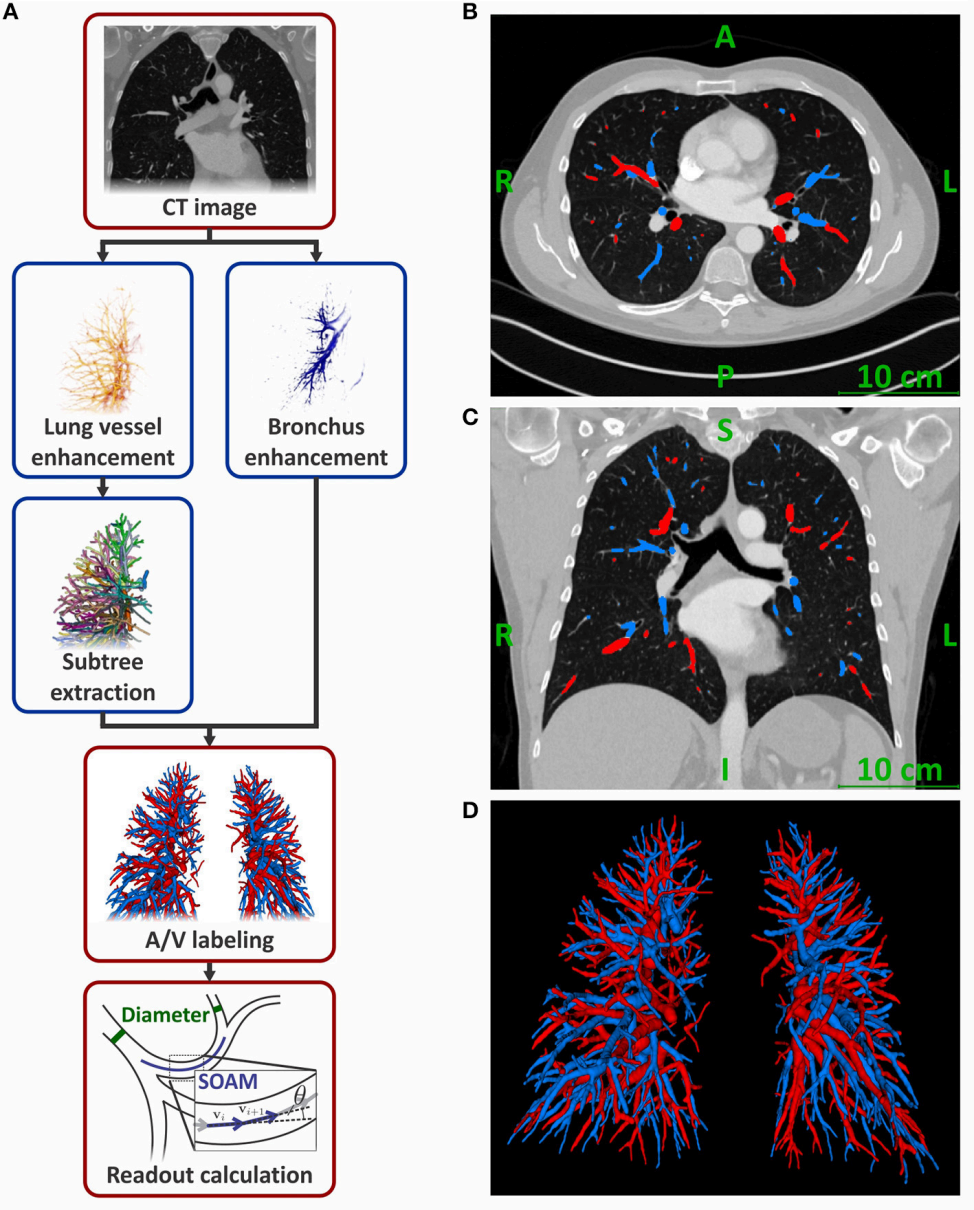
Flowchart of the fully-automatic artery/vein separation algorithm **(A)**. Representative computed tomography pulmonary angiography images in transversal **(B)** and coronal **(C)** plane of a male subject with automatically labeled arteries and veins. Representative 3D rendering of the detected vessel trees from the same subject **(D)**. Arteries are colored blue; veins are colored red.

The automatic vessel segmentation was performed by the in-house developed software as shown in Figure 1A. A detailed description and validation of the automatic vessel extraction algorithm is presented in Payer et al. (2016). Briefly, the inputs for the software are the CTPA images. After lung segmentation, a multi-scale vessel enhancement filter produces images with a high response for tubular structures together with the respective radius and a vessel orientation estimate. Regularly spaced maxima are identified in these images and connected by optimized paths following the tubular structures with sub-voxel accuracy. From these paths, the vessel trees are reconstructed and subsequently separated at the bifurcations into individual vessel segments. Only segments with diameters between 2 and 10 mm are included. Finally, the artery/vein labeling is realized by using two anatomical properties formulated mathematically as an optimization problem. First, we exploit that arteries and veins are roughly uniformly distributed in the lung. And second, we use that bronchi run approximately parallel and in close proximity to the arteries. The algorithm results in properly labeled and morphologically characterized vessel segments in most subjects (Payer et al., 2016). Besides the arterial/venous labeling, diameter, number, and tortuosity of the individual vessel segments are determined, as well as the volume of the whole arterial and venous vessel trees.

### Validation of the artery/vein separation algorithm

Detailed evaluation of the artery/vein separation algorithm showed that in few subjects the algorithm may wrongly assign all arteries and veins in whole lung lobes (Payer et al., 2016). While the readouts for the whole vasculature would still hold true, wrong labeling would average out differences between arteries and veins. Therefore, all datasets were visually inspected by a specialist in medical image processing (M.P.) in order to identify subjects in which the artery/vein labeling was inverted in larger proportions of the lungs. The relative amount of wrongly labeled vessels was estimated in each dataset. Datasets with an accuracy of the artery/vein separation below an arbitrary set threshold of 66.7% were excluded from further analysis. Corrections of the automatic assignments were not made. Validation of the artery/vein separation algorithm was performed by a radiologist (C.B.) on randomly selected datasets. All identified vessels were manually labeled as artery, vein or wrongly identified structure (e.g., bronchial walls, thickened fissures, or vessel trees where arteries and veins were fused). The overlap between automatic and manual labeling was calculated and used as quality readout. The standard deviation of the X-ray attenuation in the *musculus erector spinae* was determined in order to quantify the influence of image quality on the readouts. Likewise, the mean attenuation in the main pulmonary artery was measured to evaluate the influence of the contrast material concentration in the lung vasculature on our readouts.

### Calculation of morphological readouts

All readouts are reported for left and right lungs combined. The number of vessel segments in total and for diameter ranges between 8–10, 6–8, 4–6, and 2–4 mm were determined. These values were also normalized to the respective subject's lung volume by calculating the vessel density, enabling comparisons between subjects with different body sizes. The volume of the lung segmentation in the CTPA images was used as lung volume. Further, the cumulative volumes of the segmented arteries, veins, and the combined vessel trees were normalized to the subject's lung volume, yielding the normalized vessel volumes for arteries, veins, and all vessels, respectively. The tortuosity of the vessel segments was determined using the sum-of-angles metric (SOAM) and the distance metric (DM) (Bullitt et al., 2003). For the calculation of the SOAM, points with 1 mm distance from each other were identified along the optimized vessel path of each vessel segment. These were used as start and end points of linear sections. SOAM was calculated by summing up the angles in radian between adjacent sections of a vessel segment and dividing the result by the length of the vessel segment (Figure 1A and Supplementary Materials). DM was calculated as ratio of the length along the center line over the distance between the start and end point of the vessel segment. It is reported in % elongation compared to a straight segment by subtracting 1.0 from the ratio and multiplying it by 100%.

### Statistical analysis

Statistical analysis was performed in SPSS (Version 24.0.0.0, IBM Corp. Chicago, IL, USA) and GraphPad Prism (Version 5.04, GraphPad Software Inc., La Jolla, California). Agreement between visual inspection and the manual labeling of the artery/vein separation was analyzed using Bland-Altman analysis. The impact of gender, age, mean X-ray attenuation and vessel type (artery/vein) on artery/vein separation was analyzed using a General Linear Model (GLM). Therefore, the mentioned variables and the interaction gender*age were included in the analysis. Other readouts were tested for normality with the D'Agostino and Pearson omnibus normality test. Differences between men and women in these readouts were evaluated by *U*-test or *t*-test if they failed or passed the normality test, respectively. Likewise, data is presented as median (interquartile range) or mean ± standard deviation, if they failed or passed the normality test, respectively, unless otherwise stated. *P*-values (*p*) < 0.05 were considered statistically significant.

## Results

One hundred and thirty six subjects (men/women = 64/72) were included in this retrospective study. For each subject information on age and gender was available. CTPA examinations resulted in a mean dose-length product of 299 mGycm (230–367 mGycm) corresponding to a mean effective radiation dose of 5.7 mSv [4.4–7.0 mSv; using a standard conversion factor for chest CT of 0.019 mSv/mGycm (International Commission on Radiological Protection, 2007; Christner et al., 2010)].

### Radiological validation of the artery/vein separation algorithm

The artery/vein separation algorithm was applied to all datasets. The visual inspection of all datasets resulted in a median percentage of correctly labeled vessels of 89% (range: 32–100%). In 13 subjects, the artery/vein labeling accuracy was below 66.7% because the labeling was inverted in some lung lobes. These subjects were excluded from further analysis. From the remaining 123 subjects (men/women = 55/68) datasets of 6 men and 7 women were randomly selected according to the gender distribution and used for manual validation of the algorithm. The validation was performed by a thoracic radiologist (C.B.). The comparison between the fully automatic segmentation and the manual labeling in the 13 randomly selected subjects resulted in a median overlap of 94.6% (range: 69.2–99.9%). Bland-Altman analysis of these datasets showed that the manual labeling resulted in 3.4% higher overlap (95% confidence interval: −5.5 to 12.3%) compared to the visual inspection.

**Figure 2:**
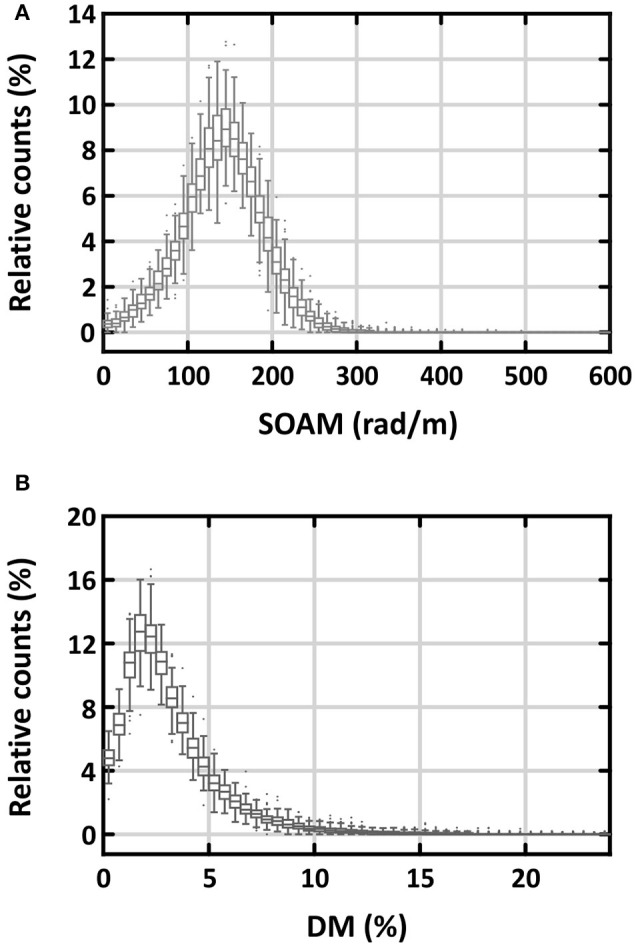
Relative distribution of tortuosity values of individual vessel segments measured with the sum-of-angles metric **(A)** and the distance metric **(B)** for all vessels. The distributions are represented as box plots of the relative amount of vessels in % of the total vessel number in both lungs for the respective tortuosity ranges. SOAM, tortuosity of vessel segments assessed by sum-of-angles metric; DM, tortuosity assessed by distance metric.

Figure 2 shows the distributions of the two measures of tortuosity. There was a significantly higher skewness of tortuosity values for the individual vessel segments for DM than for SOAM [2.19 (1.90–2.65) vs. −0.04 (−0.11–0.03), respectively; *p* < 0.001]. Further, the distribution of the DM values was very close to the minimum of 0%, which makes it hard to detect changes at this side of the distribution. Therefore, we have chosen to measure tortuosity with SOAM in this study. For comparison with other studies we present the results for DM in Supplementary Table 1.

Mean age of the subjects was 59 ± 17 years with no significant difference between men and women. The mean lung volume determined from the CTPA images was 4.6 ± 1.3 L and was significantly larger in men than in women (5.3 ± 1.3 L vs. 4.0 ± 0.9 L, respectively; *p* < 0.001). Standard deviation of the X-ray attenuation in the *musculus erector spinae* was 25 HU (19–32 HU) without a significant difference between men and women. X-ray attenuation in the main pulmonary artery was 351 HU (300–444 HU). Attenuation in women was higher than in men [359 HU (313–461 HU) vs. 336 HU (277–414 HU), respectively; *p* = 0.04]. The algorithm detected 977 vessel segments per subject (768–1,115 segments) in the diameter range of 2–10 mm. These were labeled as 487 arterial segments (390–587 segments) and 471 venous segments (371–551 segments). Representative CT images and a 3D rendering of the labeled vessels are presented in Figures 1B–D.

The results for number of vessel segments, vessel density, normalized vessel volume, and tortuosity are presented in Table 1. The analysis of these morphologic readouts was performed with a GLM considering the parameters age, gender, vessel type, and mean X-ray attenuation in the *truncus pulmonalis* at the same time. The β-values for the construction of the reference equations for the individual readouts are presented in Table 2. For clarity we discuss the influences of the individual parameters separately.

**Table 1:** Number of all vessel segments, vessel density, normalized vessel volume, and tortuosity for arteries, veins, and all vessels by gender.

		**All subjects (*n* = 123)**	**Men (*n* = 55)**	**Women (*n* = 68)**
*N* (1)	Arteries	487 (390–587)	536 (420–617)	445 (364–543)
	Veins	471 (371–551)	499 (395–562)	445 (360–527)
	All vessels	977 (768–1115)	1042 (825–1206)	867 (711–1060)
N/V_lung_ (1/L)	Arteries	106.7 (87.8–122.5)	98.1 (82.0–118.1)	111.4 (94.8–126.0)
	Veins	101.9 (84.6–119.0)	90.2 (77.7–106.7)	111.1 (95.6–122.7)
	All vessels	206.0 (181.5–237.8)	191.3 (160.6–203.0)	216.7 (192.8–260.7)
V/V_lung_ (%)	Arteries	1.52 (1.36–1.79)	1.52 (1.36–1.77)	1.53 (1.36–1.84)
	Veins	1.54 (1.37–1.71)	1.47 (1.29–1.64)	1.57 (1.41–1.75)
	All vessels	3.06 (2.72–3.47)	3.04 (2.67–3.36)	3.08 (2.75–3.57)
SOAM (rad/m)	Arteries	142.9 (139.7–146.0)	141.9 (138.3–145.8)	143.5 (140.5–146.2)
	Veins	140.6 (137.6–144.1)	140.9 (137.8–143.6)	140.4 (137.5–144.3)
	All vessels	142.1 (138.3–144.5)	140.8 (137.6–144.5)	142.7 (138.8–144.8)

*Values are given as median (interquartile range) for both lungs. N, sum of arterial and venous vessel segments; V_lung_, lung volume determined from CT image; V, volume of vessel segments with diameters between 2 and 10 mm; SOAM, tortuosity of vessel segments assessed by sum-of-angles metric*.

**Table 2:** Gender-, age-, and X-ray attenuation-dependence of number of vessel segments, vessel density, normalized vessel volume, and tortuosity for arteries, veins and all vessels.

**Reference equation:**		Y = Intercept + β_Gender_ * Gender + β_Age_ * Age + β_Age * Gender_ * Age * Gender + β_Att, mPA_ * Att_mPA_				
		**Intercept**	**β_Gender_**	**β_Age_**	**β_Age × Gender_**	**β_Att, mPA_**
N (1)	Arteries	267.30^†^	196.91^*^	1.460	−1.938	0.2636^††^
	Veins	361.33	170.12	0.986	−1.926	0.0510
	All vessels	628.62	367.02^*^	2.446	−3.864	0.3146
N/V_lung_ (1/L)	Arteries	62.16^†^	18.28	0.298	−0.508^*^	0.0924^**^
	Veins	80.01	14.00	0.194	−0.500	0.0521^†^
	All vessels	142.18	32.28	0.492	−1.008^*^	0.1445^**^
V/V_lung_ (%)	Arteries	1.52	0.33	0.004^††^	−0.006	−0.0004
	Veins	1.75	0.22	0.000	−0.006	−0.0004
	All vessels	3.27	0.55	0.004	−0.012	−0.0008
SOAM (rad/m)	Arteries	133.79	−1.75	0.021	0.019	0.0218^***^
	Veins	134.13	1.92	0.051	−0.036	0.0101
	All vessels	134.19	−0.52	0.028	0.000	0.0169, 100, 100

Values are β-coefficients of the reference equation for the respective readout. N, number of vessel segments; V_lung_, lung volume determined from CT image; V, volume of vessel segments with diameters between 2 and 10 mm; SOAM, tortuosity of vessel segments assessed by sum-of-angles metric; Y, readout of interest; Gender, men = 1/women = 0; Age, subject's age in years; Att_mPA_, mean X-ray attenuation in the truncus pulmonalis in HU; ^*^, ^**^, and ^***^: β-coefficient significantly different from 0, p < 0.05, p < 0.01, and p < 0.001; ^†^, ^††^ : significant difference between arteries and veins p < 0.05, and p < 0.01; All intercepts are significantly different from 0, p < 0.001.

### Morphologic analysis of all vessels in men and women

**Figure 3:**
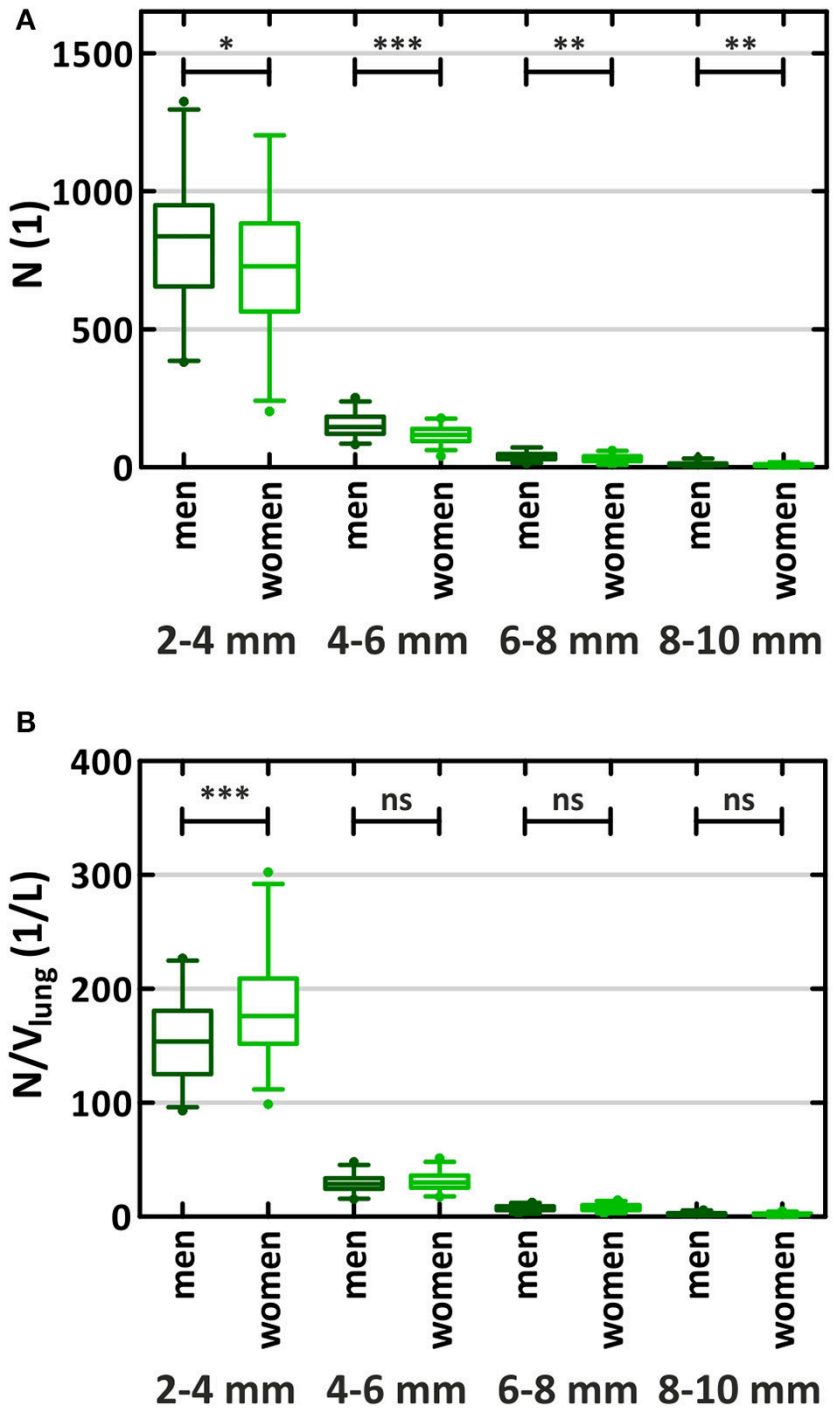
Number of vessel segments **(A)** and vessel density **(B)** by diameter and gender. *N*, sum of arterial and venous vessel segments in both lungs; V_lung_, lung volume determined from CT image; ^*^, ^**^, and ^***^: significant differences between men and women *p* < 0.05, *p* < 0.01, and *p* < 0.001; ns, not significant.

The number of vessel segments in the diameter ranges of 8–10, 6–8, 4–6, and 2–4 mm were 9 (6–12), 34 (25–44), 134 (102–159), and 797 (623–905), respectively (Supplementary Table 2). Men presented with significantly more vessel segments than women over all diameter ranges (Figure 3). This is reflected by the significant β_Gender_-value, adding 367 vessel segments for men. The vessel densities did not differ significantly between men and women, except for small vessels ranging from 2 to 4 mm diameter where men showed lower vessel densities than women [153/L (125–181/L) vs. 176/L (152–209/L), respectively; *p* < 0.001]. There was no significant offset associated with gender for normalized vessel volume. Tortuosity of all vessels was 140.8 rad/m (137.6–144.5 rad/m) in men and 142.7 rad/m (138.8–144.8 rad/m) in women and did not show a significant offset.

### Differences between arteries and veins

In the diameter range from 2 to 10 mm we detected 487 arteries (390–587 arteries) and 471 veins (371–551 veins). Vessel density was just above 100/L for both vessel types (Table 1). The total normalized vessel volume was divided into arterial and venous volume at roughly equal measures. Arteries were slightly, but not significantly, more tortuous than veins [142.9 rad/m (139.7–146.0 rad/m) vs. 140.6 rad/m (137.6–144.1 rad/m), respectively]. Arteries had significantly less vessel segments and a lower vessel density than veins (*p* = 0.011 and *p* = 0.027, respectively; Table 2). The intercept for the normalized vessel volume showed a trend to be lower in arteries than in veins (*p* = 0.052). Despite these differences, the strong influence of age and X-ray attenuation in the *truncus pulmonalis* on the respective values for arteries resulted in a virtually complete overlap between arteries and veins (Supplementary Figure 1).

### Age-dependence of lung vessel morphology

**Figure 4:**
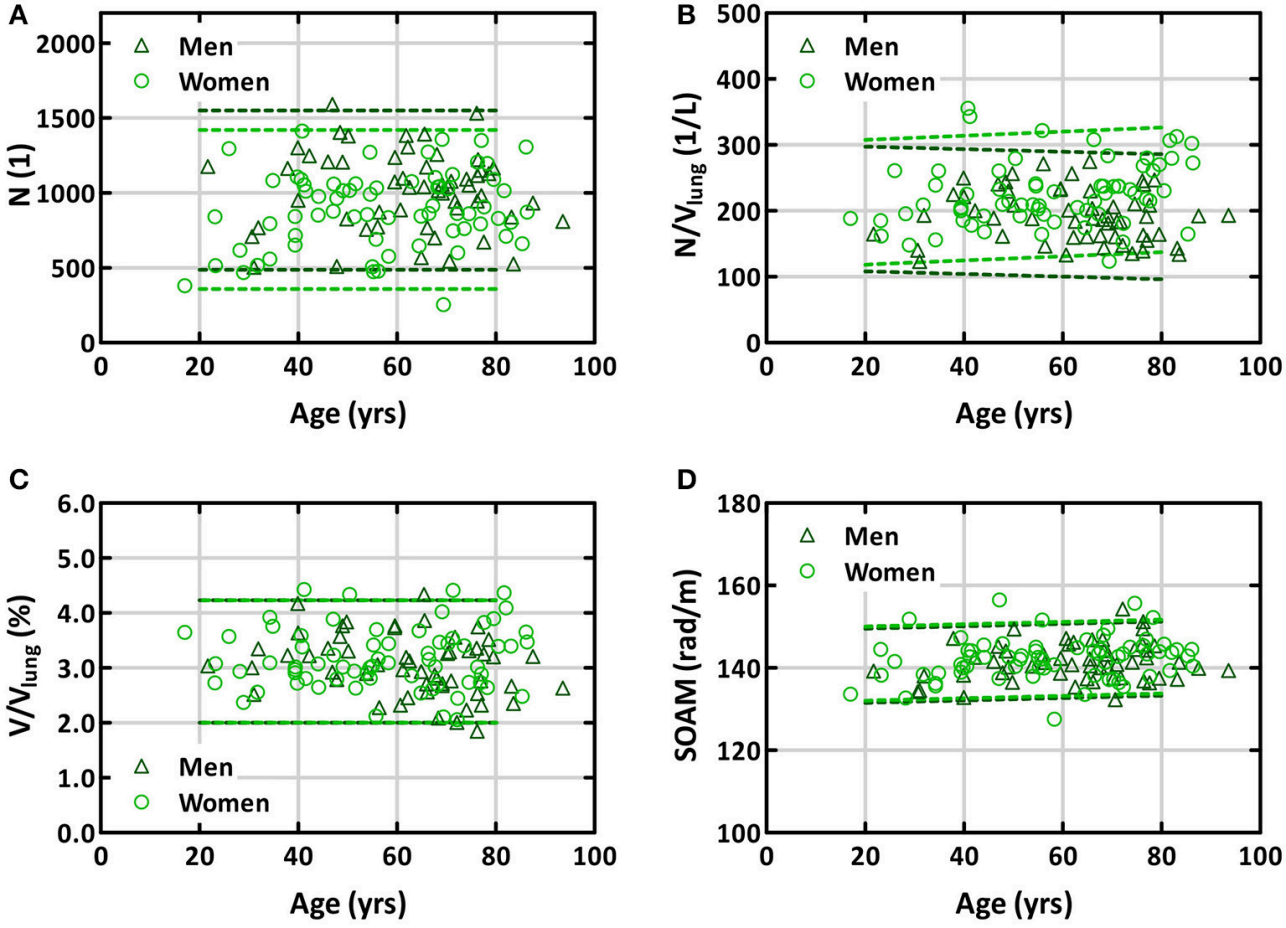
Age-relationship of number of vessel segments **(A)**, vessel density **(B)**, normalized vessel volume **(C)**, and tortuosity **(D)** for all vessels in men and women. The dashed lines represent the bands containing 95% of subjects, assuming an average X-ray attenuation in the *truncus pulmonalis* of 370 HU. *N*, sum of arterial and venous vessel segments in both lungs; V_lung_, lung volume determined from CT image; V, volume of vessel segments with diameters between 2 and 10 mm in both lungs; SOAM, tortuosity of vessel segments assessed by sum-of-angles metric.

In women, the number of all vessel segments increased with age by 2.4 vessels/year, the vessel density by 0.49/L/year, and the normalized vessel volume by 0.004%/year (Figure 4). In contrast, these readouts decreased in men by 1.4 vessels/year, 0.52/L/year, and 0.008%/year, respectively. While the gender-independent changes with age were not significant, there was a significant interaction between gender and age concerning vessel density (*p* = 0.03) and a trend concerning normalized vessel volume (*p* = 0.050; Table 2). Tortuosity was neither dependent on age for men nor for women. The only significant difference in age-dependence between arteries and veins was for normalized vessel volume (*p* = 0.001). In this readout, arteries but not veins showed a slight increase with age, although this increase was not significant by itself. The increase in tortuosity with age for arteries and veins was not significant.

### Influence of contrast material concentration in the main pulmonary artery

Increasing X-ray attenuation in the *truncus pulmonalis* resulted in an increased number of detected vessel segments and therefore also in an increased vessel density (Table 2). However, only the latter was significantly correlated with attenuation (β_Att, mPA_ = 0.1445/L/HU, *p* = 0.002). In both readouts, arteries showed a significantly stronger increase with attenuation compared to veins (*p* = 0.005 for number of vessel segments; *p* = 0.017 for vessel density). In the normalized vessel volume, we observed a weak, non-significant decrease with higher attenuation. With increasing attenuation slightly more tortuous vessels were detected (*p* = 0.001).

## Discussion

Morphometric information on the lung structures in healthy subjects is necessary to improve our understanding about the complex and interconnected functional network of the lung (Weibel, 2009, 2017). The typical methods to gain this information have been the analysis of airway and vessel casts (Singhal et al., 1973; Horsfield, 1978; Horsfield and Gordon, 1981; Huang et al., 1996) as well as the examination of tissue slides using stereology (Hsia et al., 2010). With these methods, pulmonary vessels with diameters down to 10–20 μm can be analyzed. However, they require laborious preparation procedures and are very time consuming, limiting the tractable number of samples. For instance, the analyses of the pulmonary vessel casts were performed altogether on only 12 lungs of 7 subjects (Singhal et al., 1973; Horsfield, 1978; Horsfield and Gordon, 1981; Huang et al., 1996). This illustrates the need for methods capable of analyzing larger cohorts with reasonable effort. The fully-automatic algorithm allowed characterization of arteries and veins from 2 to 10 mm diameter in 123 subjects. Furthermore, the algorithm allowed for the direct comparison between the arteries and veins in the same subject. This was not possible in previous studies on vessel casts, as arterial and venous vessel trees obtained from different subjects were analyzed (Singhal et al., 1973; Horsfield, 1978; Horsfield and Gordon, 1981; Huang et al., 1996). Using CT images also provides physiologic conditions. For comparison, the filling pressures of the vessel casts were 25 mmHg (Huang et al., 1996) and 26 mmHg (Horsfield, 1978; Horsfield and Gordon, 1981), which are conditions representing mild pulmonary hypertension for both arterial and venous casts (Kovacs et al., 2009; Hoeper et al., 2013). These pressures are expected to dilate all the vessels in the casts, particularly the larger ones.

We found that the number of detected vessels was strongly related to the lung volume. The normalized number of vessels larger than 2 mm in diameter was about 200 per liter lung volume. The number comprised about 100 arteries/L and 100 veins/L. These numbers were independent of age; however, they were about 15% higher in women than in men. This may have resulted, in part, from the higher pulmonary artery attenuation in women observed in the analyzed datasets. Despite the higher vessel density in women, the larger lung volumes in men resulted in more vessel segments over all diameter ranges. Since the anatomy of the lobar and segmental lung vessels is the same for men and women, the smaller vessels in women are subsequently counted in smaller diameter ranges than the anatomically comparable vessels in men. This shift can explain the lower number of vessel segments in women.

A direct comparison to previous studies on vessel casts is only possible for the study from Huang et al., which also presents a connection matrix converting Strahler orders to vessel segments (Huang et al., 1996). They found higher number of vessel segments in the larger diameter ranges than this study, but the results are still within the 95% confidence intervals. Thus, the difference might be a result of individual variability of the two examined subjects, the increased filling pressure used for the casts or maybe due to the higher specific weight of the cast as compared to blood causing stronger gravitational effects.

The total volume of detected vessels accounted for about 3% of lung volume. This was independent of age and gender. Arteries and veins contributed to this volume by about 50% each. Notably, there was a trend toward an age-dependent decrease of the venous volume in men which might be due to age-related emphysema. Unfortunately we had no lung function data to substantiate this speculation.

To our knowledge, our study is the first to quantify the tortuosity of both arteries and veins by age and gender. We used both distance metric and SOAM to examine vessel tortuosity. Although DM has been more widely used, we found SOAM more adequate as a measure because its distribution compares to a Gaussian distribution, whereas DM is very much skewed. SOAM was about 142 rad/m, independent of age and gender in both arteries and veins. This corresponds to an angle of approximately 9° between successive 1 mm long sections along the path of the vessel segments. It should be noted that SOAM depends on the length of the individual sections used. First, longer sections would average out small kinks and turns in the vessel paths, resulting in smaller values for the metric. And second, the measure itself depends on the length of the sections. Therefore, comparisons to other datasets should account for the section length used in the analysis.

Prior to inclusion into this study, all CTPA scans were examined for artifacts or inadequate vascular enhancement. Therefore, one can assume that the clear majority of vessels are depicted correctly. Still, image noise can be a source of error in the segmentation. With increasing noise, the measure identifying tube-like structures in the CTPA images becomes weaker and the algorithm might miss small vessels. We used the standard deviation of the X-ray attenuation of the *musculus erector spinae* as a measure to assess the influence of image quality on the results. Both, number of vessel segments, and vessel density increased with increasing noise, which was driven by a small number of subjects (see Supplementary Figure 2). If noise was a relevant factor, we would expect an inverse relationship. From this we conclude that image noise did not affect the algorithm's performance to detect vessels down to our limit of detection of 2 mm diameter.

All readouts showed a large variation between the individual subjects independent of age, gender, vessel type, and X-ray attenuation in the *truncus pulmonalis*. The large variation remained even when the mixed model was used to correct for these factors (Supplementary Figure 3). Thus, we assume that the presented results are useful as reference values for morphometric analyses. Although the vessels showing the most prominent changes in lung diseases are too small to be detected with CT, several studies have shown that morphometric readouts of the lung vasculature visible on CT images change with pulmonary vascular diseases (Matsuoka et al., 2010a,b; Estépar et al., 2013; Helmberger et al., 2014; Jacob et al., 2016, 2017; Rahaghi et al., 2016a,b). These readouts are similar to the ones discussed in this study. Therefore, we expect that the readouts of this study will be significantly different from the presented reference values in pathological states. A possible application could be the improvement of sensitivity and specificity in algorithms for Computer-aided Diagnosis (CAD) for the identification of patients with chronic lung diseases. Further, the reference values may be used to refine computational models of the pulmonary circulation (Burrowes et al., 2005; Clark et al., 2011; Tawhai et al., 2011; Huang et al., 2012).

### Limitations

We took advantage of a series of CT investigations performed in a standardized way in a high-volume diagnostic center. All patients were free of acute or chronic pulmonary embolism or other abnormalities of the heart or the lung that could be detected by CT. Unfortunately we had no information on co-morbidities like lung function abnormalities, arterial hypertension, diabetes, non-thoracic cancer, etc. Therefore, we cannot exclude that such age-related factors may have influenced our morphometric results. However, such a group of patients may better represent subjects undergoing thoracic CT investigations than a highly selected population of absolutely healthy subjects. Thus, this study provides normal ranges of the morphologic readouts applicable to the identification of patients in CAD algorithms. A further limitation of this study is the limited number of vessels accessible by CT imaging and hence by the algorithm. A typical human lung includes hundreds of millions of vessels. E.g., Huang et al. found a total of 15 generations of vessels between the main pulmonary artery and the capillaries, with diameters varying from 15 to 0.02 mm (Huang et al., 1996). In our CTPA images, we detect vessels down to a diameter of 2 mm. Smaller vessels could not reliably be detected due to the partial volume effect. Since lung vascular diseases most prominently affect the small arterioles and venules with diameters between 0.05 and 1.0 mm, this limitation may reduce the diagnostic and/or prognostic value of morphologic readouts derived from CT images. Despite this fact, the above mentioned studies still demonstrated significant differences in vessel morphology between patients and controls using the larger vessels accessible to CT with similar constraints in the vessel diameters as in the present study. Further, we chose an upper limit for vessel detection of 10 mm, because in the hilar region arteries and veins run very close to each other and the fully-automatic algorithm cannot separate them reliably enough. A refinement of the algorithm to include the *truncus pulmonalis* and veins running into the left atrium is subject to future development. While the algorithm runs fully automatically, the inadequate artery/vein labeling in approximately 10% of subjects necessitates a critical inspection of the results. Regarding the current state of the art, we consider this necessary for any algorithm. We only had information on age and gender available, thus we cannot exclude influences of other parameters on our morphological readouts. This includes the degree of inspiration which influences the lung volume and, therefore, the vessel density, relative vessel volume, and likely also the vessel tortuosity. However, under the assumption that this variation is normally distributed within our large sample, we can expect that this does not have an influence on our results, but rather reflects the variation expected in clinical routine. Finally, due to radiation exposure one cannot repeatedly scan the patients. This would be necessary to determine the reproducibility of the readouts in the same subject.

## Conclusion

We report quantitative readouts of lung vessel morphology from a large cohort of subjects with no apparent thoracic abnormalities examined by means of thoracic computed tomography and analyzed by means of a fully-automatic artery/vein separation algorithm. We obtained normal values of lung vessel morphology and provide information on their variation with respect to age and gender. These data may serve as reference values for morphometric analysis and may be useful for the identification of pathologic changes in chronic lung diseases.

## Author contributions

MP: conceived the study, acquired data, designed and executed the experiments, analyzed and interpreted the data, wrote and revised the manuscript; CB, CP, and AA: executed the experiments, analyzed and interpreted the data, read and revised the manuscript; MU, RS, and AO: guided the experimental design, read and revised the manuscript; HO: guided the experimental design, analyzed and interpreted the data, read and revised the manuscript; TJ: conceived the study, acquired data, read and revised the manuscript; FM: conceived the study, acquired data, guided the experimental design, read and revised the manuscript; ZB: conceived the study, guided the experimental design, analyzed, and interpreted the data, wrote and revised the manuscript. All authors gave final approval of the manuscript to be published.

### Conflict of interest statement

The authors declare that the research was conducted in the absence of any commercial or financial relationships that could be construed as a potential conflict of interest.
