# Supplementary material for: Healthy Lung Vessel Morphology Derived From Thoracic Computed Tomography
Source: Front Physiol. 2018 Apr 10;9:346. doi: 10.3389/fphys.2018.00346 (PMC5932382; doi:10.3389/fphys.2018.00346)
Supplement: Supplementary file 1 [file Image1.pdf]

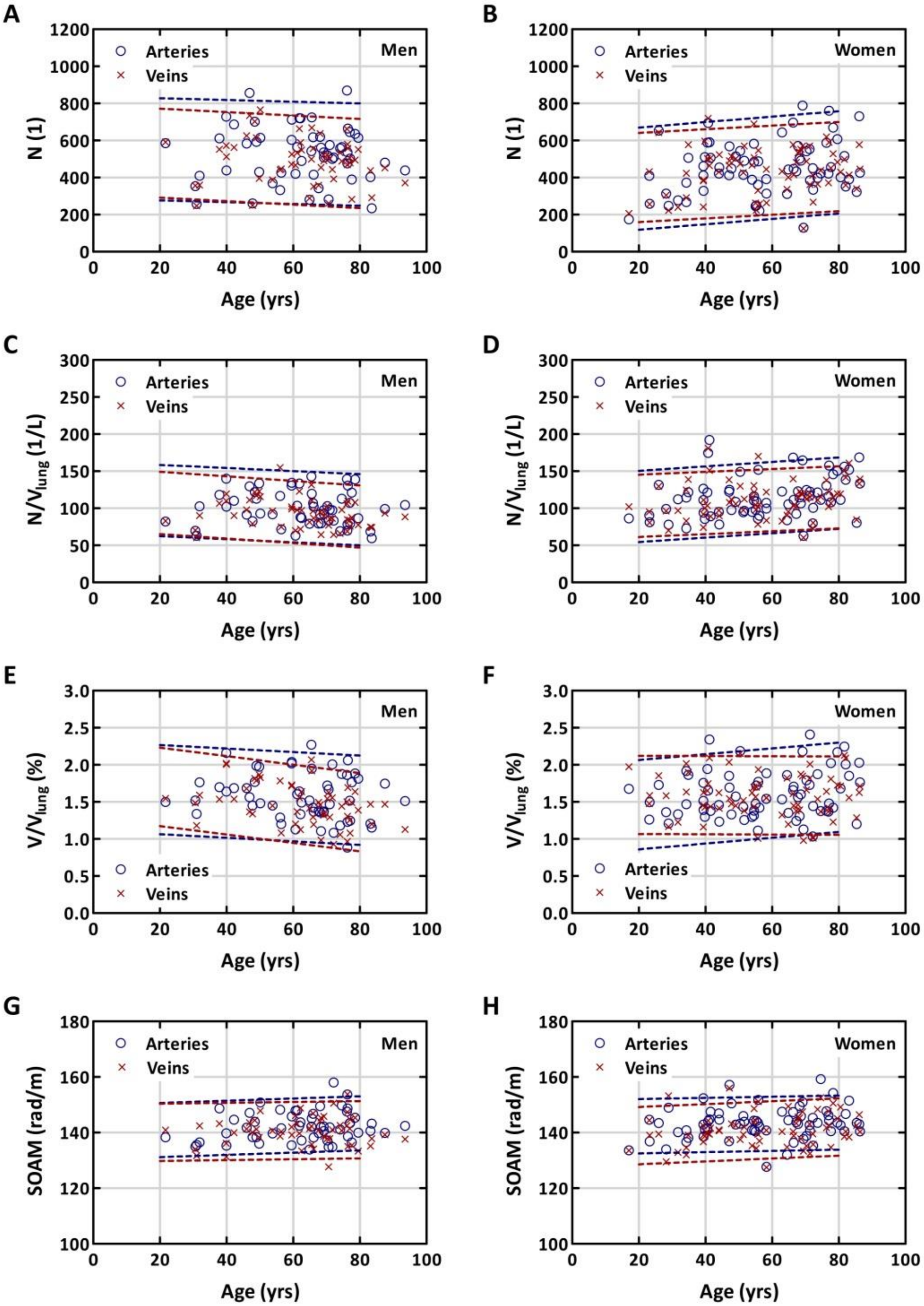

Supplementary Figure 1 | Age-relationship of number of vessel segments (A, B), vessel density (C, D), normalized vessel volume (E, F), and tortuosity (G, H) in men (A, C, E, G) and women (B, D, F, H) for arteries and veins. The dashed lines represent the bands containing 95% of subjects, assuming an average X-ray attenuation in the *truncus pulmonalis* of 370 HU. N, sum of arterial and venous vessel segments in both lungs; V_{lung}, lung volume determined from CT image; V, volume of vessel segments with diameters between 2-10 mm in both lungs; SOAM, tortuosity of vessel segments assessed by sum-of-angles metric.
