# Supplementary material for: Healthy Lung Vessel Morphology Derived From Thoracic Computed Tomography
Source: Front Physiol. 2018 Apr 10;9:346. doi: 10.3389/fphys.2018.00346 (PMC5932382; doi:10.3389/fphys.2018.00346)
Supplement: Supplementary file 2 [file Image2.pdf]

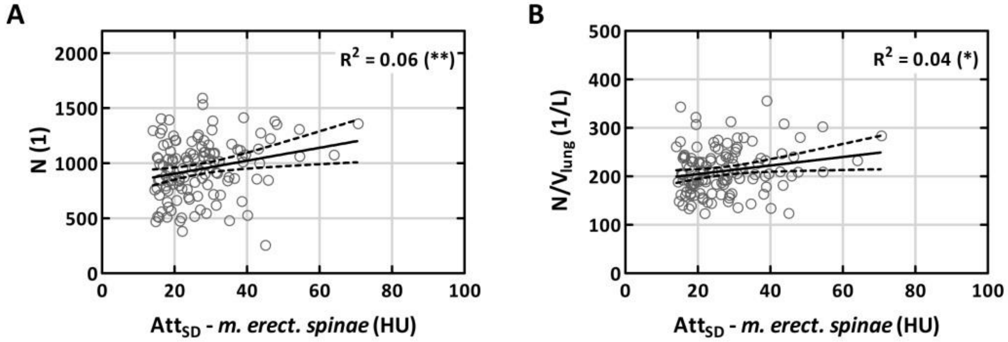

Supplementary Figure 2 | Number of vessel segments (A) and vessel density (B) over image noise assessed by standard deviation of X-ray attenuation of the *musculus erector spinae*. N, sum of arterial and venous vessel segments in both lungs; V_{lung}, lung volume determined from CT image; Att_{SD}, standard deviation of the X-ray attenuation in the *musculus erector spinae*; R^2, coefficient of determination of the linear regression; * and **: slope significantly non-zero, $p < 0.05$ and $p < 0.01$; dashed lines represent 95% confidence bands.
