# Supplementary material for: Healthy Lung Vessel Morphology Derived From Thoracic Computed Tomography
Source: Front Physiol. 2018 Apr 10;9:346. doi: 10.3389/fphys.2018.00346 (PMC5932382; doi:10.3389/fphys.2018.00346)
Supplement: Supplementary file 3 [file Image3.pdf]

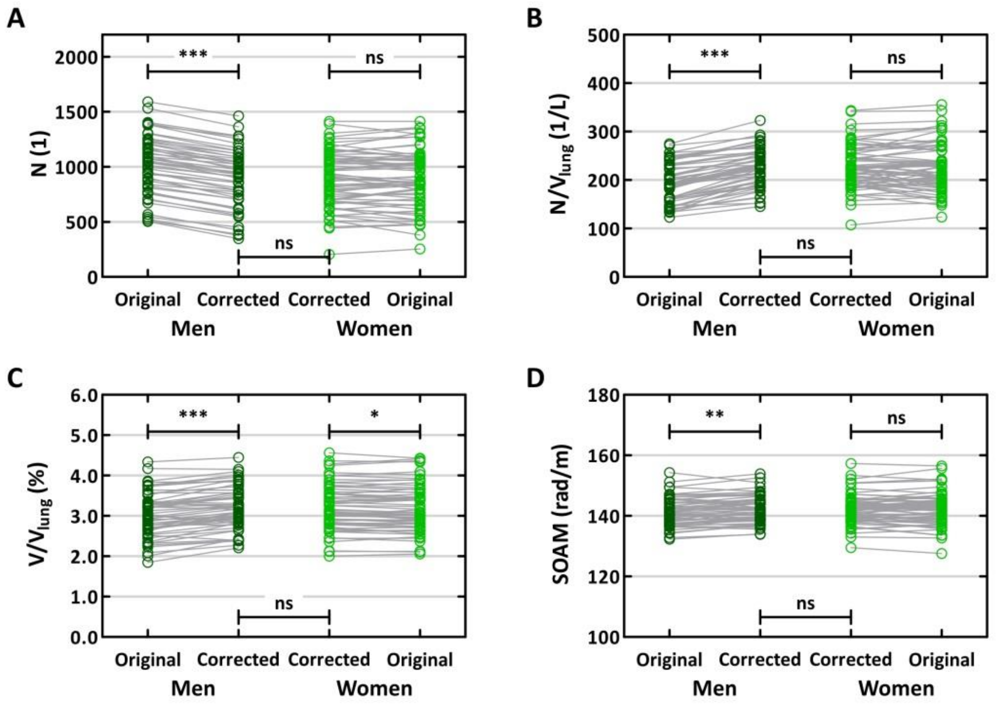

Supplementary Figure 3 | Number of vessel segments (A), vessel density (B), normalized vessel volume (C), and tortuosity (D) for men and women as measured and corrected to the average X-ray attenuation in the *truncus pulmonalis* of 370 HU, the average age of 60 years, and female gender. N, sum of arterial and venous vessel segments in both lungs; V_{lung}, lung volume determined from CT image; V, volume of vessel segments with diameters between 2-10 mm in both lungs; SOAM, tortuosity of vessel segments assessed by sum-of-angles metric; * and ***: significant differences assessed by paired and unpaired *U*-test, $p < 0.05$ and $p < 0.001$; ns, not significant.
