# Supplementary material for: Healthy Lung Vessel Morphology Derived From Thoracic Computed Tomography
Source: Front Physiol. 2018 Apr 10;9:346. doi: 10.3389/fphys.2018.00346 (PMC5932382; doi:10.3389/fphys.2018.00346)
Supplement: Supplementary file 4 [file Table1.pdf]

Supplementary Table 1 | Tortuosity for arteries, veins and all vessels by gender.

		All subjects (n=123)	Men (n=55)	Women (n=68)
**Mean DM (%)**	**Arteries**	3.20 (3.06-3.41)	3.20 (3.03-3.36)	3.19 (3.06-3.44)
	**Veins**	3.15 (3.03-3.27)	3.12 (3.02-3.19)	3.17 (3.04-3.33)
	**All vessels**	3.18 (3.04-3.32)	3.13 (3.03-3.29)	3.22 (3.07-3.37)
**Median DM (%)**	**Arteries**	2.64 (2.52-2.79)	2.61 (2.49-2.73)	2.67 (2.54-2.80)
	**Veins**	2.55 (2.47-2.65)	2.52 (2.43-2.63)	2.59 (2.49-2.66)
	**All vessels**	2.60 (2.52-2.71)	2.58 (2.48-2.69)	2.62 (2.54-2.72)

Values are given as median (interquartile range) for both lungs. DM, tortuosity of vessel segments assessed by distance metric.
