# Supplementary material for: Healthy Lung Vessel Morphology Derived From Thoracic Computed Tomography
Source: Front Physiol. 2018 Apr 10;9:346. doi: 10.3389/fphys.2018.00346 (PMC5932382; doi:10.3389/fphys.2018.00346)
Supplement: Supplementary file 5 [file Table2.pdf]

Supplementary Table 2 | Number of vessel segments and vessel density for arteries and veins by diameter and gender.

		Diameter range	All subjects (n=123)	Men (n=55)	Women (n=68)
**N (1)**	**Arteries**	8-10 mm	5 (3-7)	5 (3-8)	4 (3-6)
		6-8 mm	16 (12-23)	21 (15-27)	14 (11-20)
		4-6 mm	64 (51-83)	72 (61-92)	59 (45-70)
		2-4 mm	392 (321-476)	440 (337-509)	364 (280-434)
		Total	487 (390-587)	536 (420-617)	445 (364-543)
	**Veins**	8-10 mm	4 (2-6)	5 (3-7)	4 (2-6)
		6-8 mm	18 (12-22)	19 (13-25)	18 (11-22)
		4-6 mm	66 (55-78)	74 (57-86)	62 (52-73)
		2-4 mm	382 (307-443)	397 (326-445)	366 (283-437)
		Total	471 (371-551)	499 (395-562)	445 (360-527)
	**All vessels**	8-10 mm	9 (6-12)	10 (7-15)	8 (5-11))
		6-8 mm	34 (25-44)	41 (29-49)	31 (23-41)
		4-6 mm	134 (102-159)	146 (121-183)	118 (96-140)
		2-4 mm	797 (623-905)	836 (655-949)	728 (565-884)
		Total	977 (768-1115)	1042 (825-1206)	867 (711-1060)
**N/V_{lung} (1/L)**	**Arteries**	8-10 mm	1.1 (0.6-1.5)	1.1 (0.5-1.5)	1.1 (0.7-1.6)
		6-8 mm	3.7 (2.9-4.9)	3.8 (3.2-4.7)	3.6 (2.7-5.1)
		4-6 mm	14.1 (12.0-17.6)	14.2 (11.8-17.5)	14.1 (12.1-18.0)
		2-4 mm	84.5 (70.6-101.7)	78.9 (64.3-96.2)	90.5 (77.7-106.2)
		Total	106.7 (87.8-122.5)	98.1 (82.0-118.1)	111.4 (94.8-126.0)
	**Veins**	8-10 mm	0.9 (0.6-1.4)	0.9 (0.6-1.4)	0.9 (0.5-1.4)
		6-8 mm	3.8 (2.8-5.3)	3.6 (2.5-4.6)	4.4 (2.9-5.5)
		4-6 mm	15.1 (12.0-17.9)	14.4 (11.6-16.6)	16.1 (13.0-19.0)
		2-4 mm	80.3 (67.8-94.5)	73.7 (61.9-84.2)	87.9 (73.1-102.0)
		Total	101.9 (84.6-119.0)	90.2 (77.7-106.7)	111.1 (95.6-122.7)
	**All vessels**	8-10 mm	2.0 (1.3-2.8)	2.0 (1.3-3.0)	2.0 (1.4-2.7)
		6-8 mm	7.7 (6.0-9.8)	7.5 (6.0-9.3)	8.1 (6.0-10.3)
		4-6 mm	29.9 (24.9-34.6)	28.8 (24.4-33.7)	30.1 (25.4-36.1)
		2-4 mm	167.7 (139.1-196.3)	153.7 (125.1-181.0)	176.1 (151.9-209.3)
		Total	206.0 (181.5-237.8)	191.3 (160.6-203.0)	216.7 (192.8-260.7)

Values are given as median (interquartile range) for both lungs. N, number of vessel segments; V_{lung}, lung volume determined from CT image.
