# Supplementary material for: Healthy Lung Vessel Morphology Derived From Thoracic Computed Tomography
Source: Front Physiol. 2018 Apr 10;9:346. doi: 10.3389/fphys.2018.00346 (PMC5932382; doi:10.3389/fphys.2018.00346)
Supplement: Supplementary file 6 [file DataSheet1.pdf]

Calculation of sum-of-angles metric

The sum-of-angles metric (SOAM) was calculated following Bullitt et al. (Bullitt et al., 2003). n points (p) with 1 mm distance from each other are identified along the optimized vessel path with sub-voxel accuracy. For any point p_k the linear sections between the previous and the subsequent point define the vectors

$$v_1 = p_k - p_{k-1} \text{ and} \quad (1)$$

$$v_2 = p_{k+1} - p_k \quad (2)$$

with p_0 being the start point of the vessel path. The in-plane angle in radian between these two vectors is given by

$$\theta_k = \cos^{-1}\left(\left(\frac{v_1}{|v_1|}\right) \cdot \left(\frac{v_2}{|v_2|}\right)\right) \quad (3)$$

where $\theta_k \in [0, \pi]$ and $|v_1|$ and $|v_2|$ are the lengths of the vectors v_1 and v_2, respectively. SOAM is calculated by summing up the angles between adjacent sections of a vessel segment and dividing the result by the length of the vessel segment, which is represented by the cumulative length of the sections used:

$$SOAM = \frac{\sum_{k=1}^{n-2} \alpha_k}{\sum_{k=1}^{n-1} |p_k - p_{k-1}|} \quad (4)$$

Any remaining piece of the vessel path within 1 mm of the last point p_n was not considered in the analysis. Only vessel segments with at least two sections were analyzed.

Reference

Bullitt, E., Gerig, G., Pizer, S.M., Lin, W.L., and Aylward, S.R. (2003). Measuring tortuosity of the intracerebral vasculature from MRA images. *IEEE Trans. Med. Imaging* 22, 1163-1171. doi: 10.1109/TMI.2003.816964
